# The impacts of prenatal drought and heat stress on genetic parameter estimates for birth and weaning weights in Namibian Simmentaler and Simbra cattle

**DOI:** 10.1093/jas/skag066

**Published:** 2026-02-26

**Authors:** Sèyi Fridaïus Ulrich Vanvanhossou, Sven König

**Affiliations:** Institute of Animal Breeding and Genetics, Justus-Liebig-University Gießen, Gießen 35390, Germany; Institute of Animal Breeding and Genetics, Justus-Liebig-University Gießen, Gießen 35390, Germany

**Keywords:** genotype-by-climate interaction, environmental sensitivity, resilience, climate change, random regression, crossbreed

## Abstract

Climatic instability with recurrent drought and heat stress imposes significant constraints on beef production in Namibia. Elucidating animal genetic responses to these environmental pressures is essential for enhancing herd resilience. This study assesses genotype-by-environment interactions (GxE) under prenatal drought and heat stress on birth weight (BW) and weaning weight (WW) in Namibian Simmentaler (SM) and Simbra (SB) cattle. Four environmental conditions (EC) were defined to characterize cumulative precipitations over 365, 280, and 90 d before birth, and average temperature humidity index (THI) over 90 d before birth. The genetic parameters of the traits in each breed were modeled as functions of the EC using a bivariate reaction norm model. Estimated direct heritabilities for BW (SM: 0.22–0.43; SB: 0.35–0.52) increased gradually from drought or heat stress conditions to more favorable EC. Conversely, direct heritabilities for WW (SM: 0.10–0.36; SB: 0.25–0.47) were low under moderate conditions and high at both extremes of the EC gradients. Maternal heritabilities for BW (SM: 0.05–0.22; SB: 0.08–0.19) and WW (SM: 0.04–0.21; SB: 0.07–0.12) were consistently lower and mainly increased with improved EC. These variations in heritabilities underline reduced selection response and genetic gain under drought and heat stress. Negative direct (SM: −0.42 to 0.66; SB: −0.07 to 0.88) and maternal (SM: −0.27 to 0.32; SB: 0.31–0.94) genetic correlations $\left({rg}_{EC}\right)$ between same traits from extreme EC gradients confirm the presence of strong GxE effects due to precipitation and THI. Direct ${rg}_{EC}$ were larger for BW and lower for WW, relative to the maternal ${rg}_{EC}$, reflecting that GxE mainly influences maternal genetic effects for prenatal growth and direct genetic effects for postnatal growth. Cumulative precipitations over 365 d and THI over 90 d implied the largest GxE influences on BW and WW, respectively. Simbra showed reduced environmental sensitivity relative to SM, in line with its Brahman background. Comparisons of estimated breeding values along EC gradients evidenced the occurrence of sire re-rankings in both breeds. About 50% robust, 30% plastic, and 20% extremely plastic genotypes among the elite bulls in each breed indicate selection opportunities for robustness against time-lagged drought and heat stress. The findings highlight the significance of GxE and the potential to mitigate animal sensitivity, thereby optimizing breeding strategies in Namibian SM and SB cattle.

## Teaser

This study demonstrates the significant influence of pre-conception and late gestation environmental conditions on maternal and direct genetic effects for birth and weaning weights in Namibian Simmentaler and Simbra cattle. The findings reveal opportunities to select for resilience to drought and heat stress, thereby optimizing breeding and selection strategies in Namibian beef cattle.

## Graphical Abstract

**Graphical Abstract:**
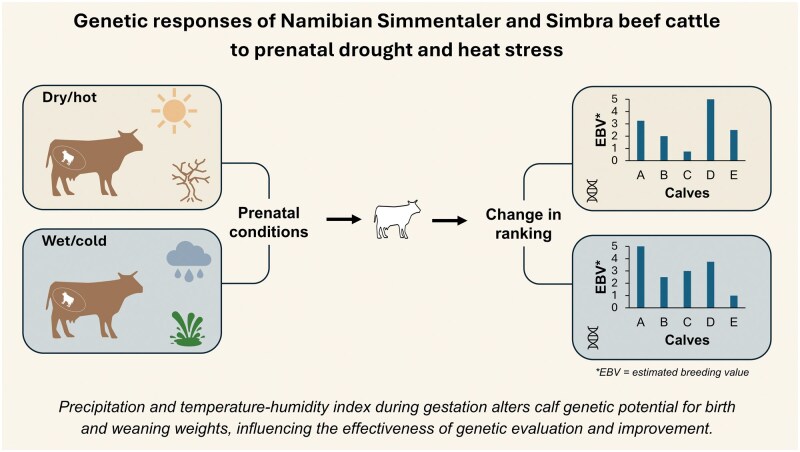

## Introduction

Semi-arid and unstable climatic conditions pose significant challenges to the Namibian beef industry (Vallejo Orti and Negussie 2019). Beef farming in the country mainly relies on extensive grazing systems, wherein cattle are kept year-round on natural rangelands. In this context, prolonged intra-seasonal droughts, which have become increasingly frequent in recent years, impair the availability and quality of grasses (Awala et al. 2021; Cooke et al. 2025). Simultaneously, rising temperatures in the region cause heat stress and exacerbate the vulnerability of animals to diseases (Kaurivi et al. 2021). These climatic pressures have detrimental effects on herd productivity, leading to reduced animal growth performance and increased mortality, with significant economic implications (Thuiller et al. 2006). Farmers are therefore constantly required to react and adapt to their stressful and precarious production environment (Lohmann et al. 2014).

Understanding how animals respond to diverse environmental stressors is crucial for adopting resilient breeding strategies. The concept of genotype-by-environment interactions (GxE) refers to when individual genotypes exhibit variable responses to different environmental conditions (EC) (Silva Neto et al. 2024). Concretely, changes in breeding values or low correlations (below 0.8) of genetic effects across environments indicate the presence of GxE (Falconer 1990). Genotype-by-environment interaction effects are associated with the adaptation of an animal or a breed to a production environment, especially in the context of harsh environmental influences. For instance, Burrow (2012) noted important re-ranking of sires across environments in breeds poorly adapted to tropical climate. In Namibia, as in many beef production systems, breeding animals are produced on elite or nucleus farms, and afterwards, used on commercial farms, reflecting a variety of production systems. This implies that a bull selected in a favorable environment, as well as its offspring, could later present limited performances under stressful EC, or vice versa, when GxE is ignored. Information on GxE effects is therefore crucial to identify relevant testing environments, the most appropriate genetic materials for specific production environments, or more adapted genotypes that perform well in diverse environments. Such information will ensure the optimization of resource allocation and selection efficiency in breeding programs (de Leon et al. 2016; de Paula Freitas et al. 2021).

The Simmentaler breed (official name of Simmental cattle in Namibia) and its derived crossbred Simbra (Simmentaler × Brahman) are very popular beef cattle breeds in Namibia. Originated from Europe, Simmentaler cattle were introduced to Namibia in 1893 (Massmann 2020). Simbra cattle, governed by a structured breeding society with an open herdbook policy, have flexible breed compositions tailored to environmental constraints and breeders’ objectives. Both breeds are reported for remarkable production performances and adaptability to the arid climate in the country (Coleman 2018; Gouws 2021). However, the genetic responses of Namibian Simmentaler and Simbra cattle in relation to their fluctuating EC have not yet been evaluated. Studies in the U.S. and Brazil, where Simmentaler were similarly introduced, have observed evidence of GxE, especially for growth traits (Braz et al. 2021; Moura et al. 2023). Moreover, heat stress and suboptimal nutritional conditions during gestation often cause significant alterations of genetic components, compromising fetal development and subsequent production performances of beef calves (Tao and Dahl 2013).

The current study aims to: 1) investigate the impacts of heat stress and prenatal drought (at different time periods during gestation) on the genetic (co)variance components and breeding values for birth and weaning weights (WW) of offspring in Namibian Simmentaler and Simbra cattle; 2) infer the magnitude of GxE in the breeds due to variations in prenatal EC.

## Materials and methods

### Animal data

This study involved a total of 17,369 Simmentaler and 22,897 Simbra beef cattle from Namibia. Phenotypic and pedigree data from existing routine records were provided by the Simmentaler Simbra Cattle Breeder’s Society of Namibia; therefore, no ethical approval was required. The animals were born between 1986 and 2024 and kept in 17 farms distributed in five vegetation zones in Namibia (Jarvis et al. 2022). Detailed information and structure of the data for each breed are presented in Table 1. Additional statistics of pedigree structure and quality are summarized in Table S1.

**Table 1:** Data structure for birth weight (BW) and weaning weights (WW) in Namibian Simmentaler and Simbra beef cattle.

	Simmentaler			Simbra		
	BW	WW	Total	BW	WW	Total
**Records, *n***	14,280	10,370	15,254	16,922	16,497	19,983
**Animals in pedigree data, *n***	16,668	12,949	17,369	2,0295	19,515	22,897
**Contemporary groups, *n***	620	546	635	347	372	391
**Farms, *n***	12	12	12	5	5	5
**Vegetation zones, *n***	4	4	4	3	3	3
**Mean, kg**	38.64	234.31	**—**	34.86	226.96	**—**
**SD, kg**	5.09	42.80	**—**	4.95	42.74	**—**

Traits of interest included birth weight (BW, kg) and WW (kg, measured between 150- and 300-d post-partum). Phenotypic values outside the range of mean ±3.5 standard deviation units were set as missing. Data filtering excluded all animals with an unknown birth date or missing both BW and WW information. Contemporary groups were formed by concatenating the farm, animal birth year and season, and only contemporary groups with more than five observations were considered. Birth seasons were defined as early rainy season (October to December), late rainy season (January to April), early dry season (May to July), and late dry season (August and September). We used this classification to reduce intra-seasonal variability, accounting for differences in rainfall patterns, pasture conditions, and temperature across the year (Du Plessis 2001; Cooke et al. 2025).

### Weather data and EC

A total of four EC characterizing prenatal drought and heat stress were evaluated for Simmentaler and Simbra animals in Namibia. Time-lagged prenatal drought conditions, defined as sumPrec365, sumPrec280, and sumPrec90 were calculated for each animal by summing up daily precipitations during 365, 280, and 90 d, respectively, before its birth date. We focused on these periods because of their importance in fetal development and following previous publications (Mayer and Musani 2002; Halli et al. 2021; Alves et al. 2022). Daily precipitations between 1986 and 2024 were retrieved from the Climate Hazards Center InfraRed Precipitation with Station (CHIRPS) data (Funk et al. 2015) based on the farm geographical coordinates and using the Google Earth Engine platform. The CHIRPS data provides long-term gridded daily precipitation information with a 0.05° resolution (approximately 5 km) and is suitable for assessing drought in Southern Africa (Du Plessis and Kibii 2021; Du et al. 2024).

Moreover, the average temperature humidity index (THI) over the 90 d before the birth date (meanTHI90) were calculated as EC for heat stress. For this purpose, we retrieved hourly air temperature and dewpoint temperature at 2 m above the surface from the ERA5-Land (Muñoz-Sabater et al. 2021). The ERA5-Land provides publicly available long-term gridded climate data with a resolution of 9 km and constitutes a reliable alternative for weather station data, especially in tropical areas (Tan et al. 2023). The calculation of THI applied the formula (equation 1) proposed by the National Research Council (1971), as it is known to adequately measure heat stress for outdoor cattle (Bohmanova et al. 2007).


(1) THI=(1.8 T+32)-(0.55-0.0055RH)×(1.8 T-26)

where THI was the temperature humidity index; T was the air temperature; RH was the relative humidity from the August-Roche-Magnus formula (Alduchov and Eskridge 1996; Westermann et al. 2016; equation 2);


(2) $$RH=\frac{\;(\frac{e^{\left(17.625\;\times DT\right)}}{243.04\;\times DT})\;}{\;(\frac{e^{\left(17.625\;\times T\right)}}{243.04\;\times T})\;}\times 100$$

where DT was the dewpoint temperature, and T was the air temperature.

### Statistical analyses

All statistical analyses in this study were performed separately for each breed.

First, we assessed the effects of the EC (i.e., sumPrec365, sumPrec280, sumPrec90, meanTHI90) on BW and WW phenotypes by applying a univariate linear mixed model with the *lmer* function of the R package *lme4* (Bates et al. 2015). The statistical model (equation 3) was defined as follows:


(3) $$y_{\mathrm{ijklmn}}=\mu +{Sb}_{j}+P_{k}+{Vz}_{l}+{b_{1}Aw}_{i}+b_{2}{EC}_{i}+D_{m}+{Cg}_{n}+e_{\mathrm{ijklmn}}$$

where $y_{\mathrm{ijklmn}}$ was BW or WW of $i^{th}$ animal; μ was the overall mean; ${Sb}_{j}$ was the fixed effect of the $j^{th}$ animal sex and birth type (four classes: pairwise combinations of females and males with singleton and twin births); $P_{k}$ was the fixed effect of the $k^{th}$ dam parity (1 to 7, all animals with parity > 6 were assigned to class 7); ${Vz}_{l}$ was the fixed effect of the $l^{th}$ vegetation zone (five classes), $b_{1}$ was the linear regression coefficient of age at weaning on WW of $i^{th}$ animal (${Aw}_{i}$); $b_{2}$ was the linear regression coefficient of EC (${EC}_{i}$) on BW or WW of $i^{th}$ animal; $D_{m}$ was the random maternal effects of the $m^{th}$ dam$;{Cg}_{n\;}$was a random effect of $n^{th}$ contemporary groups (see definition above), and $e_{\mathrm{ijklmn}}$ was the random residual effect.

Second, bivariate pedigree-based reaction norm models (RNM) were applied to infer the effects of each EC on genetic parameters and breeding values of BW and WW in Namibian Simmentaler and Simbra beef cattle. The bivariate RNM (equation 4) was defined as follows:


(4) $$[\begin{array}{c} y_{1}\\ y_{2} \end{array}]=[\begin{array}{c} X_{1}a_{1}+Q_{1}b_{1}+R_{1}c_{1}+Z_{1}u_{1}+\;W_{1}v_{1}+e_{1}\\ X_{2}a_{2}+Q_{2}b_{2}+R_{2}c_{2}+Z_{2}u_{2}+\;W_{2}v_{2}+e_{2} \end{array}]\;$$

where $y_{1}$ and $y_{2}$ was BW and WW, respectively, $a_{1}$ and $a_{2}$ were vectors of the fixed effects including sex and birth type, parity, vegetation zones, linear regression coefficient of age at weaning on the WW, and the Legendre polynomials fixed regression coefficients (intercept and slope) of EC on BW and WW respectively, $b_{1}$ and $b_{2}$ were vectors of random effects of contemporary groups for BW and WW respectively; $c_{1}$ and $c_{2}$ were vectors of random effects of maternal permanent environmental for BW and WW, respectively; $u_{1}$ and $u_{2}$ were vectors of Legendre polynomials random regression coefficients (intercept and slope) of EC on the direct additive genetic effects for BW and WW respectively, $v_{1}$ and $v_{2}$ were vectors of Legendre polynomials random regression coefficients (intercept and slope) of EC on the maternal additive genetic effects for BW and WW respectively; the $X_{1},\;X_{2},\;Q_{1},\;Q_{2},\;R_{1},\;R_{2},\;Z_{1},\;Z_{2},\;W_{1},\;W_{2}$ were the respective incidence matrices; $e_{1}$ and $e_{2}$ were vectors of residuals effects for BW and WW, respectively. The first-order Legendre polynomial covariates related to EC (for fixed and random regressions) were defined as 1 (intercept) and scaled EC (slope) between −1 and 1 based on the minimum and maximum of the respective EC (see Table S2).

The covariance structure of random effects was assumed as:


(5) $$\mathrm{Var}[\begin{array}{c} \mu \\ \nu \\ \mathrm{Mpe}\\ \mathrm{Cg}\\ e \end{array}]=[\begin{array}{ccccc} A\;⨂{\;G}_{\mu }\; & A\;⨂\;G_{\mu \nu } & 0 & 0 & 0\\ \;A\;⨂\;G_{\mu \nu } & A\;⨂\;G_{\nu } & 0 & 0 & 0\\ 0 & 0 & I_{m}\;⨂{\;P}_{m}\; & 0 & 0\\ 0 & 0 & 0 & I_{g}\;⨂\;C_{g}\; & 0\\ 0 & 0 & 0 & 0 & \;I_{n}\;⨂\;R\; \end{array}]$$

were $G_{\mu }$, $G_{\nu },$ and $G_{\mu \nu }$ were 4 × 4 variance—covariance matrices of intercept and slope for BW and WW due to direct additive genetic, maternal additive genetic and covariance between direct and maternal genetic effects, respectively; A was the pedigree-based relationship matrix; $P_{m}$ and $C_{g}$ were 2 × 2 variance—covariance matrices between BW and WW due to the maternal permanent environmental and contemporary group effects, respectively; $I_{m}$, $I_{g}$, and $I_{n}$ were identity matrices for the maternal permanent environmental (of size m × m, with m = number of dams), for the contemporary group effects (of size g × g with g = number of contemporary groups) and for the residuals (of size n × n with n = number of animals), respectively; ⨂ was the Kronecker product between matrices, R was the 2 × 2 variance-covariance matrix of residual effects between BW and WW.

Estimates of the variance components from the bivariate RNM were derived by fitting the Expectation-Maximization Restricted Maximum Likelihood procedure as implemented in BLUPF90+ (Misztal et al. 2014).

Direct heritability ($h_{d}^{2}$) and maternal heritability ($h_{m}^{2}$) at a specific EC gradient were calculated for each trait as follows:


(6) $$h_{d}^{2}=\frac{\sigma_{\mu }^{2}}{\sigma_{\mu }^{2}+\sigma_{\nu }^{2}+\sigma_{\mu \nu }+\sigma_{mpe}^{2}+\sigma_{cg}^{2}+\sigma_{e}^{2}}\;$$

(7) $$h_{m}^{2}=\frac{\sigma_{\nu }^{2}}{\sigma_{\mu }^{2}+\sigma_{\nu }^{2}+\sigma_{\mu \nu }+\sigma_{mpe}^{2}+\sigma_{cg}^{2}+\sigma_{e}^{2}}$$

were $\sigma_{\mu }^{2}$ and $\sigma_{\nu }^{2}$ were the variances of direct and maternal genetic effects, respectively; $\sigma_{\mu \nu }$ was the covariance between direct and maternal genetic effects; $\sigma_{\mathrm{mpe}}^{2}$, $\sigma_{cg}^{2},$ and $\sigma_{e}^{2}$ were the variances for maternal permanent environmental effect, contemporary groups effects and residuals, respectively.

The correlation ($r_{EC}$) between the genetic effects (direct or maternal) for a trait at two different EC gradients (a and b) was calculated as follows:


(8) $$r_{EC(a,b)}=\frac{\sigma_{\left(a,\;b\right)}}{\sqrt{\sigma_{\left(a\right)}^{2}.\sigma_{\left(b\right)}^{2}}\;}$$

where $\sigma_{\left(a,\;b\right)}$ was the covariance between the genetic effects (direct or maternal) at the EC gradients a and b; $\sigma_{a}^{2}$ and $\sigma_{b}^{2}$ were the genetic variances (direct or maternal) at EC gradients a and b, respectively.

The direct or maternal genetic correlation between BW and WW at a specific EC gradient was calculated as:


(9) $$r_{bw-ww}=\frac{\sigma_{bw-ww}}{\sqrt{{\sigma_{bw}^{2}.\sigma }_{ww}^{2}}\;}$$

where $\sigma_{bw}^{2}$ and $\sigma_{ww}^{2}$ were the direct or maternal genetic variances of BW and WW, respectively, and $\sigma_{bw-ww}$, their covariance.

The correlations ($r_{\mu_{0}\mu_{1}}$) between $\mu_{0}$ (intercept of the direct additive genetic effects in the reaction norm reflecting baseline performance) and $\mu_{1}$ (slope of the direct additive genetic effects in the reaction norm reflecting environmental sensitivity) was calculated as in equation 10.


(10) $$r_{\mu_{0}\mu_{1}}=\frac{\sigma_{\mu_{0}\mu_{1}}}{\sqrt{{\sigma_{\mu_{0}}^{2}.\sigma }_{\mu 1}^{2}}\;}$$

where $\sigma_{\mu_{0}}^{2}$ and $\sigma_{\mu_{1}}^{2}$ was the variance of $\mu_{0}$ and $\mu_{1}$, respectively, and $\sigma_{\mu_{0}\mu_{1}}$ the respective covariance.

Moreover, we calculated the slope-to-intercept variance ratio ($\sigma_{\mu_{1}}^{2}/\sigma_{\mu_{0}}^{2}$) to quantify the magnitude of genetic variation in environmental sensitivity (slope) relative to baseline performance (intercept).

To evaluate the impact of G × E on Namibian Simmentaler and Simbra cattle, we examined the rankings of elite sires (i.e., sires with a minimum of 20 progenies) along the EC gradients based on their estimated direct breeding values (EBV). Direct EBV at a specific EC gradient were computed for each trait as:


(11) $$EBV_{i}=\mu_{0i}+(\mu_{1i}\times \overset{-}{EC})\;$$

where ${\mathrm{EBV}}_{i}$ was the estimated breeding value of $i^{th}$ animal at EC gradient, $\overset{-}{EC}\;$was the scaled EC between −1 and 1, and $\mu_{0i}$ and $\mu_{1i}$ were the intercept and slope, respectively, for the direct genetic effects for $i^{th}$ animal.

Furthermore, we applied the methodology by Mattar et al. (2011) to classify the phenotypic plasticity of elites sires based on the absolute value of their respective $\mu_{1}$, with $\left|\mu_{1i}\right|$ < $\sigma_{\mu_{1}}$ = robust genotype (no phenotypic differences in response to environmental variations); $\sigma_{\mu_{1}}\leq \;|\mu_{1i}|$ < ${2\sigma }_{\mu_{1}}$= plastic genotype (phenotypic differences in response to environmental variations); and $|\mu_{1i}|\geq \;2\sigma_{\mu_{1}}$ = extremely plastic genotype (substantial phenotype differences in response to environmental variations), where $\sigma_{\mu_{1}}$was the standard deviation of $\mu_{1}$ for all animals.

## Results

### EC and their effects on cattle weight phenotypes

Table 2 reports correlations between the EC for Namibian Simmentaler and Simbra cattle. Correlations between sumPrec365, sumPrec280, and sumPrec90 ranged between 0.24 and 0.68. In contrast, MeanTHI90 was negatively correlated with sumPrec365 and sumPrec280. The descriptive statistics for the EC in the two breeds are presented in Figure S1 and Table S2.

**Table 2:** Correlations between environmental conditions in Namibian Simmentaler (above diagonal) and Simbra (below diagonal) beef cattle.

Environmental conditions^1^	sumPrec365	sumPrec280	sumPrec90	meanTHI90
**sumPrec365**	1	0.65	0.28	−0.05
**sumPrec280**	0.68	1	0.43	−0.35
**sumPrec90**	0.24	0.38	1	0.34
**meanTHI90**	−0.27	−0.54	0.36	1

1 sumPrec365, sumPrec280, sumPrec90 = cumulative precipitation over 365, 280, and 90 d before birth; meanTHI90 = average temperature-humidity index over 90 d before birth.

The average BW and WW of Simmentaler calves were 38.64 ± 5.09 kg and 234.31 ± 42.80 kg, respectively. In comparison, Simbra calves recorded lower averages of BW (34.86 ± 4.95 kg) and WW (226.96 ± 42.74 kg). The results of the linear regression analyses, describing the impacts of the EC on animal weights at the phenotypic scale, are summarized in Table 3. SumPrec365 and sumPrec90 conditions presented significant effects (*P *≤ 0.005) on both BW and WW, irrespective of the breed. In contrast, improved sumPrec280 was significantly associated only with increase in Simmentaler WW (*P *< 0.001). An increase of meanTHI90 was significantly associated with a decrease of WW (*P *< 0.001) but displayed no significant effect on BW in the two breeds.

**Table 3:** Summary of linear regression analyses of calf birth weight (BW) and weaning weight (WW) phenotypes on environmental covariates in Namibian Simmentaler and Simbra beef cattle.

Environmental covariates^1^	Simmentaler				Simbra			
	BW		WW		BW		WW	
	Coef.	*P-*value	Coef.	*P*-value	Coef.	*P*-value	Coef.	*P*-value
**sumPrec365**	0.002	<0.001	0.038	<0.001	0.003	<0.001	0.053	<0.001
**sumPrec280**	0.0003	0.489	0.028	<0.001	0.001	0.272	0.005	0.268
**sumPrec90**	0.004	<0.001	−0.018	0.005	0.003	<0.001	−0.054	<0.001
**meanTHI90**	0.057	0.001	−0.991	< 0.001	0.042	0.01	−1.454	<0.001

1 sumPrec365, sumPrec280, sumPrec90 = cumulative precipitation over 365, 280, and 90 d before birth; meanTHI90 = average temperature-humidity index over 90 d before birth.

### Heritabilities and variances components along environmental gradients

**Figure 1:**
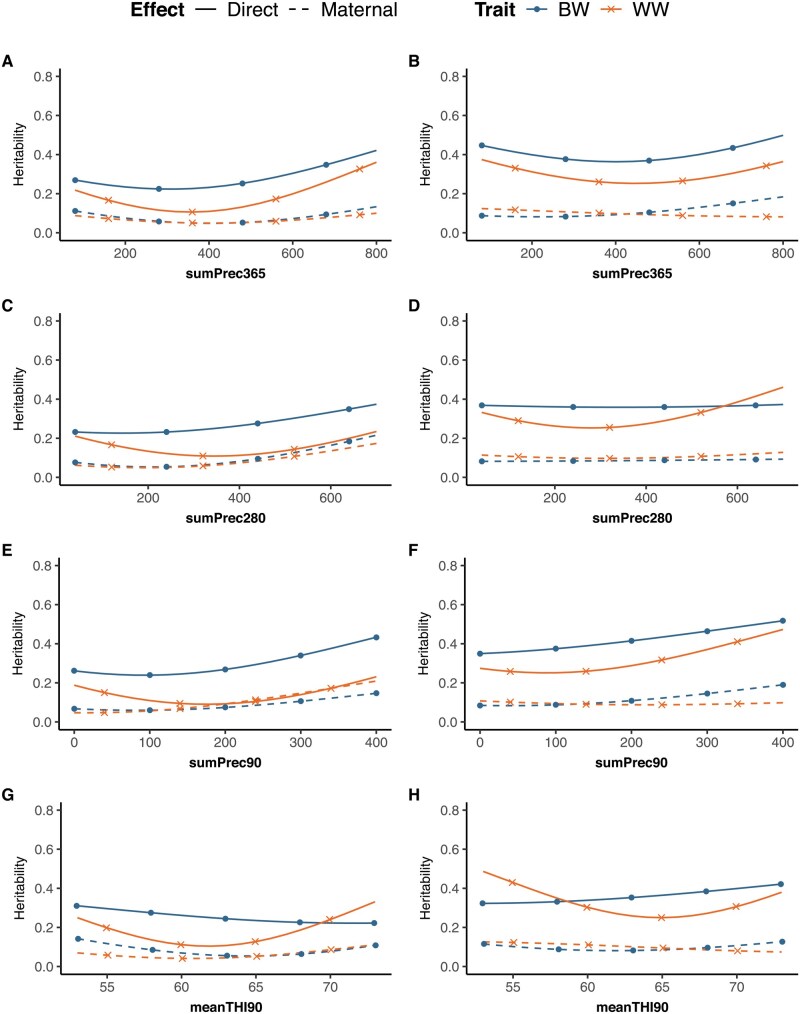
Direct and maternal heritability estimates for birth weight (BW) and weaning weight (WW) according to prenatal environmental conditions in Namibian Simmentaler (left; A, C, E, G) and Simbra (right; B, D, F, H) beef cattle. sumPrec365, sumPrec280, sumPrec90 = cumulative precipitation over 365, 280, and 90 d before birth; meanTHI90 = average temperature-humidity index over 90 d before birth.

As shown in Figure 1, direct and maternal heritability estimates for BW and WW exhibited considerable variations along most EC gradients, irrespective of the breed. BW had larger direct heritability than WW across most environments, whereas maternal heritabilities were comparable. All heritability estimates had small standard deviations below or equal to 0.05.

In the Simmentaler breed, the direct heritability for BW (0.22–0.43) increased gradually from harsh to more favorable environments, especially with increasing sumPrec280 and sumPrec90, and declining meanTHI90. In contrast, the direct heritabilities for WW (0.10–0.36) were lowest in moderate conditions and increased in both harsh and favorable environments, irrespective of the EC. Maternal heritabilities for BW (0.05–0.22) and WW (0.04–0.21) were consistently lower than the respective direct heritabilities, and they mainly increased with greater sumPrec280 and sumPrec90 gradients, respectively. SumPrec365 induced the largest variation of direct heritabilities for BW and WW. Furthermore, meanTHI90 mainly implied variation in the direct heritability for WW.

Direct heritabilities in the Simbra breed (BW: 0.35–0.52; WW: 0.25–0.47) were generally higher than those in the Simmentaler. The direct heritabilities in Simbra increased gradually with increasing sumPrec90 but were low at moderate sumPrec365 conditions and high at the extremes. The maternal heritability for BW (0.08–0.19) increased in response to increasing sumPrec90 and sumPrec365 values, while maternal heritabilities for WW (0.07–0.12) were almost constant along the EC. SumPrec280 gradients implied no variation in the heritability estimates in Simbra, except for the direct heritability for WW. The variability for the direct heritability was most pronounced with regard to sumPrec90 for BW and with meanTHI90 for WW. A rise in meanTHI90 led to a larger direct heritability for BW, whereas the direct heritability for WW decreased from meanTHI90 values 53 to 65 and slightly increased afterwards.

Changes in direct additive genetic variances ($\sigma_{\mu }^{2}$**)** and maternal additive genetic variances ($\sigma_{\nu }^{2}$) along the EC gradients were comparable to those of the respective heritabilities (see Figure S2). Variances of contemporary groups ($\sigma_{\mathrm{cg}}^{2}$), maternal permanent environments ($\sigma_{\mathrm{mpe}}^{2}$), and residuals ($\sigma_{e}^{2}$) are presented in Table S3. Variability due to the contemporary groups contributed to a large proportion of the total phenotypic variance and was more pronounced for WW (Simmentaler: 39%–53%, Simbra: 33%–47%) than for BW (Simmentaler: 15%–20% Simbra: 7%–10%). Proportions of $\sigma_{\mathrm{mpe}}^{2}$ to $\sigma_{p}^{2}$ were lower compared to $\sigma_{\mathrm{cg}}^{2}$, ranging for BW between 3%–4% in the two breeds, and for WW between 6%–8% in Simmentaler and 3%–4% in Simbra.

### Genetic correlations between the same traits along environmental gradients

**Figure 2:**
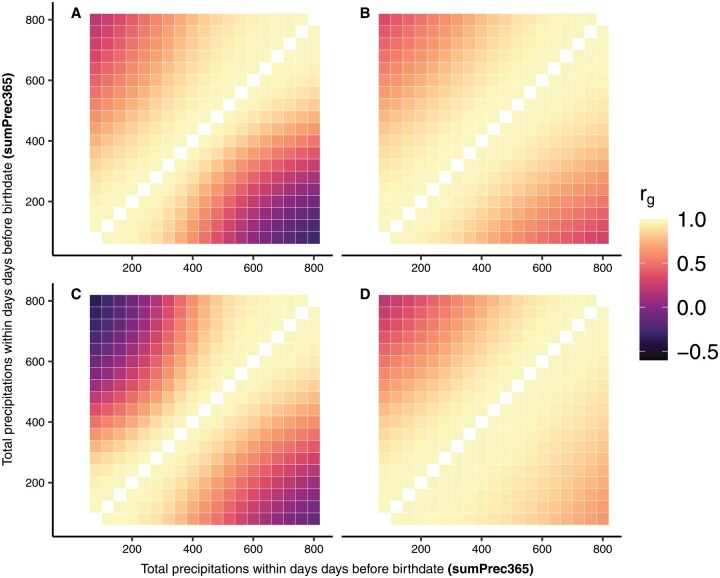
Genetic correlations (direct = upper diagonal, maternal = lower diagonal) between gradients of cumulative precipitation over 365 days before birth (sumPrec365) for birth weight (top; A, B) and weaning weight (bottom; C, D) in Simmentaler (left; A, C) and Simbra (right; B, D) cattle.

**Figure 3:**
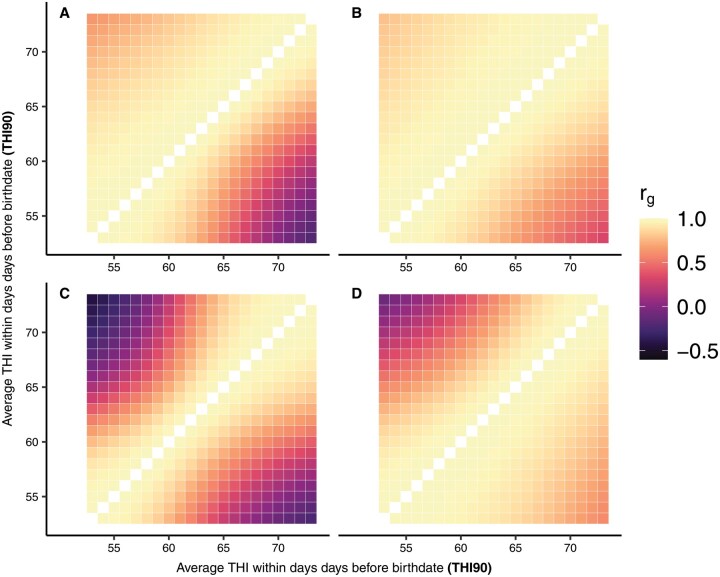
Genetic correlations (direct = upper diagonal, maternal = lower diagonal) between gradients of average temperature-humidity index over 90 days before birth (meanTHI90) for birth weight (top; A, B) and weaning weight (bottom; C, D) in Simmentaler (left; A, C) and Simbra (right; B, D) cattle.

The heatmaps in Figures 2 and 3. display variations in direct ${rg}_{EC}$ (upper diagonal) and maternal ${rg}_{EC}$ (lower diagonal) for the two traits along gradients of sumPrec365 and meanTHI90, respectively. Changes in ${rg}_{EC}$ along gradients of sumPrec280 and sumPrec90 are reported in Figures S3 and S4. Both direct and maternal ${rg}_{EC}$ decreased significantly with increasing distances between EC, and reached the lowest values between extreme gradients of EC. Negative ${rg}_{EC}$ were observed between some EC gradients. Direct ${rg}_{EC}$ were larger for BW and lower for WW, when compared to the maternal ${rg}_{EC}$, reflecting that GxE mainly influenced maternal genetic effects for prenatal growth, whereas they were more pronounced on direct genetic effects for postnatal growth. The largest declines in direct and maternal ${rg}_{EC}$ in the Simmentaler breed were observed with regard to sumPrec365 for BW (direct ${rg}_{EC}\;$≥ 0.21; maternal ${rg}_{EC}$ ≥ −0.27, Figure 2) and meanTHI90 for WW (direct ${rg}_{EC}$≥ −0.42; maternal ${rg}_{EC}$ ≥ −0.21, Figure 3). Simbra animals displayed larger ${rg}_{EC}$ than the Simmentaler, with the lowest values for BW between extreme SumPrec365 (direct ${rg}_{EC}$≥ 0.36; maternal ${rg}_{EC}$ ≥ 0.31, Figure 3). Regarding WW, the lowest direct ${rg}_{EC}\;$(≥ −0.07) was observed with regard to meanTHI90, and the lowest maternal ${rg}_{EC}\;$(≥ 0.40) with regard to SumPrec90. Direct and maternal ${rg}_{EC}$ for BW were consistently high along SumPrec280 (direct ${rg}_{EC}$≥ 0.88; maternal ${rg}_{EC}$ ≥ 0.94), indicating no evidence of GxE on BW due to SumPrec280 variations in the Simbra population.

### Genetic correlations between BW and WW along environmental gradients

**Figure 4:**
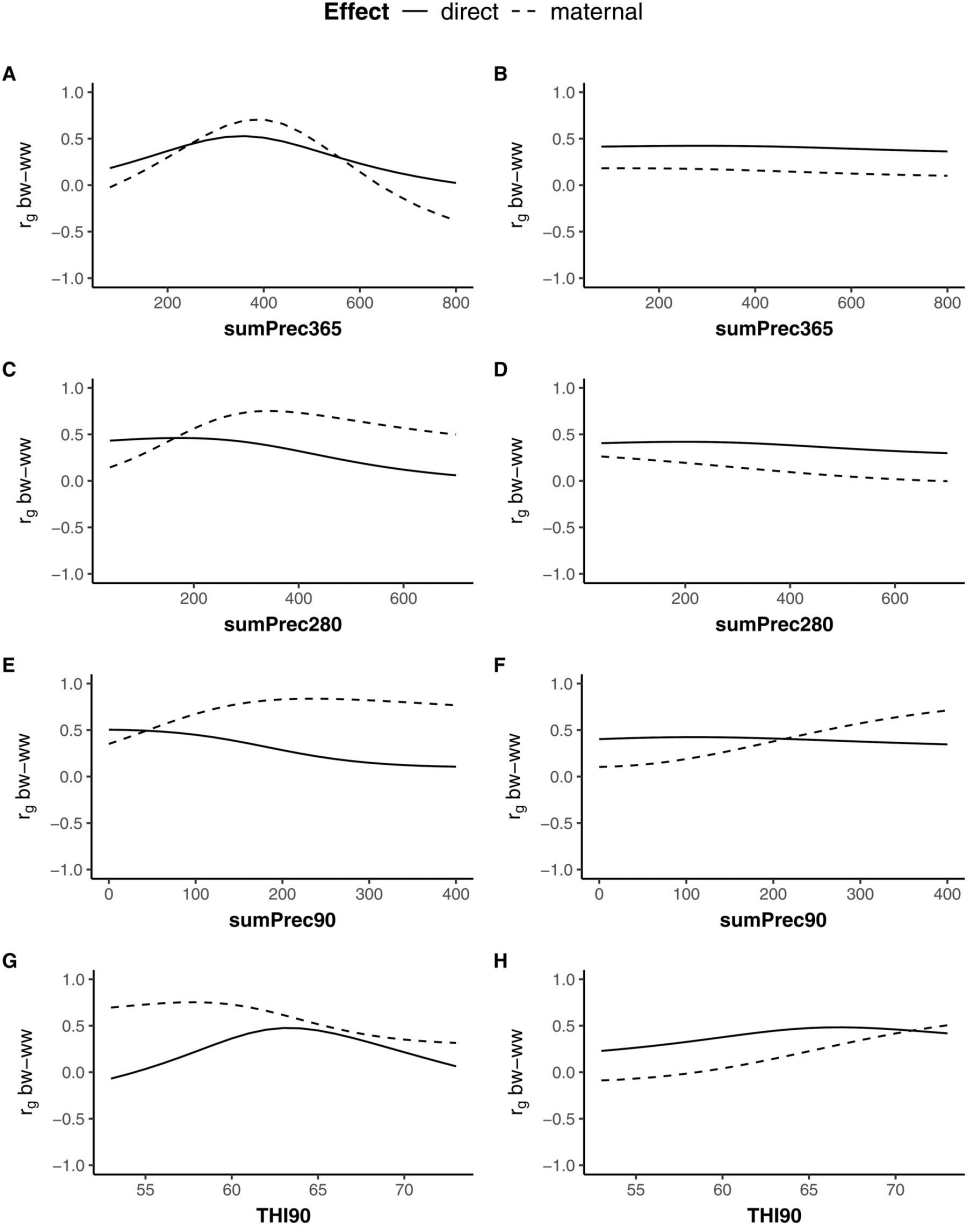
Direct and maternal genetic correlations between birth weight (BW) and weaning weight (WW) according to prenatal environmental conditions in Namibian Simmentaler (left; A, C, E, G) and Simbra (right; B, D, F, H) beef cattle. sumPrec365, sumPrec280, sumPrec90 = cumulative precipitation over 365, 280, and 90 d before birth; meanTHI90 = average temperature-humidity index over 90 d before birth.

Variations in direct and maternal genetic correlations between BW and WW (${rg}_{bw-ww}$) along the EC gradients are presented in Figure 4. In the Simmentaler breed, increasing precipitations as well as extreme meanTHI90 (low and high values) led to a decline in direct ${rg}_{bw-ww}$ (−0.09 to 0.53). Conversely, maternal ${rg}_{bw-ww}$ (−0.37 to 0.84) increased to their maximum and remained stable when sumPrec280 and sumPrec90 conditions were moderate to high. Negative direct ${rg}_{bw-ww}$ were observed at low meanTHI90 values, and negative maternal ${rg}_{bw-ww}$ at high sumPrec365 values. Direct ${rg}_{bw-ww}$ (0.23–0.48) in Simbra animals were relatively stable, with most increase along meanTHI90. Maternal ${rg}_{bw-ww}$ (−0.09 to 0.71) were similarly stable along sumPrec365 and sumPrec280 but increased significantly with increasing sumPrec90 and meanTHI9.

### Parameters related to environmental sensitivity and patterns of reaction norms

Slope-to-intercept variance ratios for direct genetic effects ($\sigma_{\mu_{1}}^{2}/\sigma_{\mu_{0}}^{2}$) were large in the two breeds, with WW exhibiting the highest values (see Table 4). Simmentaler animals (BW: 0.23–0.63; WW: 1.30–2.05) showed significantly greater ratios $\sigma_{\mu_{1}}^{2}/\sigma_{\mu_{0}}^{2}$ than Simbra (BW: 0.06–0.48; WW: 0.54–1.17). The largest ratios $\sigma_{\mu_{1}}^{2}/\sigma_{\mu_{0}}^{2}$ for BW and WW were observed with regard to sumPrec365 and meanTHI90, respectively. Moreover, $r_{\mu_{0}\mu_{1}}$ were low to moderate for most of the EC (Table 4). Lower $r_{\mu_{0}\mu_{1}}$ were observed in Simmentaler (BW: −0.29 to 0.37; WW 0.10 to 0.33) in comparison to Simbra (BW. −0.04 to 0.46; WW: −0.21 to 0.40). Negative $r_{\mu_{0}\mu_{1}}$ were mainly observed with regard to meanTHI90 for BW and WW in Simmentaler and Simbra, respectively. The highest $r_{\mu_{0}\mu_{1}}$ for BW and WW in Simmentaler were obtained with regard to sumPrec280 and sumPrec365, respectively, whereas sumPrec90 presenting the highest $r_{\mu_{0}\mu_{1}}$ in Simbra. Slope-to-intercept variance ratios and $r_{\mu_{0}\mu_{1}}$ related to maternal genetic effects are presented in Table S4.

**Table 4:** Genetic correlation ($r_{\mu_{0}\mu_{1}}$) between intercept and slope for direct additive genetic effects for birth weight (BW) and weaning weight (WW) from the reaction norm model, and slope-to-intercept variance ratio ($\sigma_{\mu_{1}}^{2}/\sigma_{\mu_{0}}^{2}$) describing environmental sensitivity to precipitation and average temperature-humidity index conditions in Namibian Simmentaler and Simbra beef cattle.

Environmental covariates^1^	Simmentaler		Simbra	
	BW	WW	BW	WW
$r_{\mu_{0}\mu_{1}}$				
** sumPrec365**	0.27	0.33	0.03	−0.01
** sumPrec280**	0.38	0.10	−0.04	0.24
** sumPrec90**	0.37	0.17	0.46	0.40
** meanTHI90**	−0.29	0.18	0.35	−0.21
$\sigma_{\mu_{1}}^{2}/\sigma_{\mu_{0}}^{2}$				
** sumPrec365**	0.68	2.05	0.48	0.65
** sumPrec280**	0.33	1.30	0.06	0.76
** sumPrec90**	0.50	1.57	0.14	0.54
** meanTHI90**	0.23	2.42	0.13	1.17

1 sumPrec365, sumPrec280, sumPrec90 = cumulative precipitation over 365, 280, and 90 d before birth. meanTHI90 = average temperature-humidity index over 90 d before birth.

**Figure 5:**
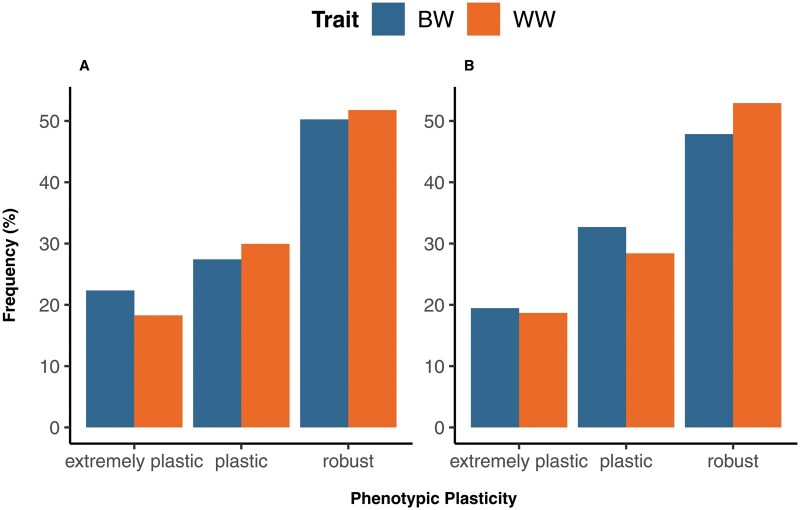
Distribution of phenotypic plasticity in birth weight and weaning weight along gradients of cumulative precipitation over 365 d before birth among elite sires (with at least 20 progeny) of Namibian Simmentaler (left; A) and Simbra (right; B) beef cattle.

**Figure 6:**
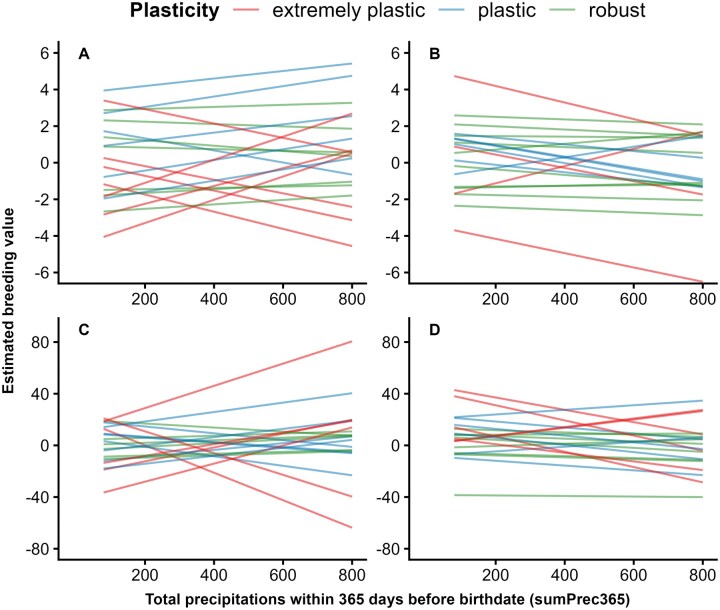
Reaction norms of the 20 sires with most progenies for birth weight (top; A, B) and weaning weight (bottom; C, D) along gradients of cumulative precipitation over 365 d before birth in Namibian Simmentaler (left; A, C) and Simbra (right; B, D) beef cattle.

The analysis of the reaction norm coefficients for direct genetic effects revealed different patterns of phenotypic plasticity within each population, as summarized in Figure 5. Irrespective of breed and trait, approximately 50% of elite bulls were classified as robust, 30% as plastic and 20% as extremely plastic in response to variations in sumPrec365. Moreover, 30% of the elite bulls showed robust genotypes with regard to sumPrec365 for BW and WW simultaneously (see Figure S5). Evidence of sire re-ranking within the top 20 elite sires (with the most progeny) is illustrated in Figure 6. Similar patterns of phenotypic plasticity and reaction norms were observed along the other EC, as presented in Figures S6, S7, and S8.

## Discussion

### Time-lagged impacts of prenatal drought and heat stress

The observed BW and WW for Simmentaler and Simbra cattle are comparable to values previously reported for these breeds in the literature (Vega-Murillo et al. 2012; Braz et al. 2021; Göncü and Sucak 2024). The results of the univariate linear regression analyses showed that higher precipitation and lower THI conditions are significantly associated with improved BW and WW phenotypes in Simbra and Simmentaler beef cattle. Also, the genetic parameters of these traits exhibited large variation in response to the change in the EC. These findings highlight the significant influence of EC both during and before gestation on growth performances in the investigated populations. Precipitation and THI conditions are important environmental stressors in beef cattle as they are related to feed availability and quality as well as disease pressures (Collier et al. 2019). The results for sumPrec90 and meanTHI90 support previous reports on the impacts of prenatal nutritional environment and heat stress in calf performance (Alves et al. 2022; Halli et al. 2021; Hay and Roberts 2018). The last trimester before calving is of common interest when investigating the effects of maternal EC on calf weight traits, as researchers observed most fetal growth during this period (Tao and Dahl 2013). Nonetheless, the results for sumPrec365 prove that long exposure of a cow to drought during the period before conception also induced significant detrimental impacts on calf growth performances. This finding is in line with results by Mayer and Musani (2002), who observed significant effects of total rainfall one year before calving on cow milk performances. Drought during the last trimester before conception (as considered with sumPrec365) could imply forage scarcity and feed restriction until the first period of gestation, especially in the semi-arid context of Namibia where animals mainly rely on pasture. In this regard, evidences in the literature have demonstrated that feed restriction or heat stress at both during the pre-conception and early gestation periods influenced fetal and postnatal development (Caton et al. 2020; Olson et al. 2021; Costa et al. 2022). The fact that precipitation is associated with forage availability at a subsequent period may provide a rationale for the limited effects of sumPrec280 on the growth traits and some genetic parameters. These observations corroborate previous reports that cows and embryos develop increased resilience to environmental stress at a mid-stage of gestation (Ealy et al. 1993; Whitney et al. 2009; Castagnino et al. 2015). Overall, the differences in the effects of sumPrec280 relative to sumPrec365 and sumPrec90 suggest that sumPrec280 captures environmental influences (particularly feed availability) during mid-gestation, which appear less critical than conditions during pre-conception and early gestation (sumPrec365) and late gestation (sumPrec90).

The large variations in heritabilities and additive genetic variances reflect differential genetic responses due to gestational heat stress and drought. Concretely, the increase in direct heritabilities for BW with improved EC gradients indicates the potential for enhanced response to selection and maximal genetic gain for BW under favorable precipitation and THI conditions in Namibian Simmentaler and Simbra. This trend is consistent with previous findings in Brazilian Simmental cattle (heritability for yearling weight ranging from 0.33 in the worst to 0.51 in the best environments, Moura et al. 2023) as well as other studies focusing on BW in beef cattle (e.g., Santana et al. 2013). In contrast to BW, the direct heritabilities for WW generally decreased in moderate conditions and increased in extreme conditions. These results suggest that postnatal decline of maternal effects makes genetic differences in calves under extreme EC more apparent, illustrating pronounced GxE. Santana et al. (2013) observed similar differences between BW and WW in terms of heritability alterations along environmental gradients. Moreover, the observed U-shaped trend of heritabilities for WW is consistent with previous results, especially those that evaluated GxE effects due heat stress (Hay and Roberts 2018; Halli et al. 2021).

The variations in maternal heritabilities along the EC gradients confirm the importance of maternal genetic effects in shaping calf performance, especially under environmental stress (Ferrell 1993). Low maternal heritabilities for BW at low EC gradients indicate that drought and heat stress reduce the ability of Simmental and Simbra cows to fully express their genetic potential, which consequently affects fetal growth. Moreover, the high correlation between maternal genetic effects for BW and WW at improved sumPrec280 and sumPrec90 (${rg}_{bw-ww}$ > 0.8) highlights that the genetic determinants of maternal nurturing ability and milk production, relevant for intrauterine and postnatal calf development, are shared and highly susceptible to environmental factors (Bradford et al. 2016; Toghiani et al. 2020).

### Magnitude of genetic-by-environment interactions and environmental sensitivity

The decline of most direct and maternal ${rg}_{EC}$ below 0.8 with the increase in EC confirm the presence of GxE effects on BW and WW due to drought and heat stress in Namibian Simmentaler and Simbra cattle. More specifically, negative ${rg}_{EC}$ at some extreme EC are indicative of a high degree of GxE. These results corroborate the reports of GxE for growth traits in Simmental and Brahman crosses in the U.S. and in Brazil (Thrift et al. 2000; Braz et al. 2021; Moura et al. 2023). The observed ${rg}_{EC}$ value of −0.42 for Simmentaler WW is lower than most genetic correlations reported in previous studies (Silva Neto et al. 2024). To the best of our knowledge, only Alves et al. (2022) observed a lower ${rg}_{EC}$ of −0.49 for BW with regard to the genetic response of Brazilian Angus-Brangus cattle to heat stress. Saavedra-Jiménez et al. (2013) reported a genetic correlation of −0.36 between dry tropic and wet tropic for WW in Mexican Braunvieh, a value comparable to the direct ${rg}_{EC}$ of −0.35 obtained with regard to sumPrec365 in the Namibian Simmentaler. With regard to BW, lower maternal ${rg}_{EC}$ than direct ${rg}_{EC}$ imply larger influences of GxE on maternal genetic effects, and corroborate the adaptive ability of the uterine environment to limit effects of adverse environments on fetal growth (Dimasuay et al. 2016; Sferruzzi-Perri et al. 2023). Conversely, more pronounced GxE on direct genetic effects of WW (lower direct ${rg}_{EC}$ than maternal ${rg}_{EC}$) suggest that calves genes involved in postnatal growth are significantly altered by environmental stresses occurring during gestation, despite protective maternal influences.

The analysis of reaction norms patterns substantiates the consequences of GxE in Namibian Simmentaler and Simbra cattle, providing evidence that animals with high genetic merit for BW or WW under favorable precipitations and THI conditions do not necessarily maintain their superiority under drought and heat stress. Occurrences of sire re-rankings, as illustrated in Figure 6 and Figures S6–S8, concur well with the observed low to moderate correlations ($r_{\mu_{0}\mu_{1}}$). Similar values of $r_{\mu_{0}\mu_{1}}$ were reported by Alves et al. (2022) and Santana et al. (2025) regarding heat stress and pasture conditions, respectively. The observations of large ratios of $\sigma_{\mu_{1}}^{2}/\sigma_{\mu_{0}}^{2}$ suggest that genetic variations in Namibian Simmentaler and Simbra cattle animal performances is primarily attributable to differences in environmental sensitivity, rather than the baseline genetic merit. This implies that selection responses or genetic improvements for growth traits critically depends on the sensitivity of the animals to drought and heat stress (Kolmodin and Bijma 2004). The ratios $\sigma_{\mu_{1}}^{2}/\sigma_{\mu_{0}}^{2}$ for BW are comparable to those reported for BW in Brazilian Hereford-Braford and Angus-Brangus (Alves et al. 2022) as well as for yearling weight in Nellore cattle (Santana et al. 2025). In contrast, the ratios $\sigma_{\mu_{1}}^{2}/\sigma_{\mu_{0}}^{2}$ of 2.05 and 2.42 for Simmentaler WW with regard to sumPrec365 and meanTHI90, respectively, were substantially larger than those from the literature. The findings that sumPrec365 and meanTHI90 exhibited the lowest ${rg}_{EC}$ and largest ratios $\sigma_{\mu_{1}}^{2}/\sigma_{\mu_{0}}^{2}$ for BW and WW, respectively, indicate that variations in these EC imply the strongest impacts on the genetic effects of the traits (Kolmodin and Bijma 2004). More remarkably, the results with regard to meanTHI90 suggest that postnatal growth in the investigated populations are more sensitive to heat stress than to drought. This observation may be related to the extended effects of heat stress on both calves and cows, affecting their feed intake, metabolic rate, health as well as milk production (Tao and Dahl 2013).

### Breed differences

The larger maternal heritabilities and ${rg}_{bw-ww}$ in Simmentaler cattle relative to Simbra, indicate that Simmentaler dams contribute more substantially to the genetic merit of the calves than Simbra cows. This observation aligns with the significant protective maternal influences against environmental stress (as evidenced through the negative ${rg}_{EC}$) particularly observed in Simmentaler cattle. Brandt et al. (2010) observed a comparable strong mothering character and a maternal heritability of 0.18 for BW in German Simmental beef cattle. Similarly, maternal heritabilities for WW under moderate to high precipitation environments in this study are consistent with the estimate of 0.14 reported by Hendriks et al. (2022) in South African Simmentaler cattle under feedlot conditions. Our estimates for the direct heritabilities for BW and WW, particularly under moderate to high precipitation conditions, exceeded the estimates previously reported for other Simmentaler populations (ranging from 0.14 to 0.23 for BW and from 0.12 to 0.15 for WW; Glaze et al. 1994; Brandt et al. 2010; Amaya-Martínez et al. 2019). These discrepancies suggest a greater genetic potential of Namibian Simmentaler cattle but may also result from the use of a RNM in the current study. By partitioning genetic effects along environmental gradients, RNMs better capture additive genetic variances while reducing the residual error term, thereby yielding higher heritability estimates (Schaeffer 2004; Pedrosa et al. 2023). Moura et al. (2023) applied a RNM and similarly reported higher direct heritabilities (0.33–0.51) for yearling weight in Brazilian Simmental cattle.

Larger direct heritabilities in Simbra compared to Simmentaler reflect the genetic contribution of the Brahman breed to their genome. Our results are consistent with previous heritabilities estimates in South African Simbra and other Brahman-derived crossbreds, such as Hereford-Braford and Angus-Brangus in Brazil (Schoeman et al. 2000; Alves et al. 2022). Also, the reduced sensitivity to drought and heat stress in Simbra compared to Simmentaler cattle is in agreement with findings by van der Nest et al. (2020), who detected selection signatures related to environmental adaptability and productivity in the South African population. Williamson and Humes (1985) associated larger BW in Brahman crosses with their comparatively longer gestation time, which may enable a prolonged adaptive time to environmental stress. The hardiness and enhanced adaptation of Brahman and its crosses to tropical environments may explain the observed increase in direct heritabilities with increasing meanTHI90 in Simbra cattle. These observations suggest that Simbra calves tend to be more comfortable at larger THI, contrasting with the findings in Simmentaler and other European-origin beef cattle (Halli et al. 2021).

### Implications for resilient breeding strategies

This study highlights the critical role of GxE in shaping the genetic architecture of growth traits in Namibian Simmentaler and Simbra cattle. Variations of the genetic parameters along the EC gradients imply that accounting for environmental sensitivity in breeding programs is essential for enhancing resilience and sustaining genetic progress in the instable Namibian beef production climatic context (Mulder 2016; Oliveira et al. 2018). Significant GxE affecting direct genetic effects of WW alongside limited maternal influences suggests that this trait could be more appropriate for assessing direct growth tolerance to drought and heat stress. Bradford et al. (2016) similarly identified WW as a suitable trait for developing adaptive strategies to heat stress in Angus cattle. In addition, accounting for maternal robustness in breeding goals improves calf adaptability, long-term genetic improvement, and financial performance of beef farms (Kelly et al. 2021). Considering the pronounced effects of sumPrec365 and meanTHI90 on the growth trait variations, the integration of environmental covariates reflecting pre-conception and prenatal conditions into genetic evaluations can assist in modeling GxE and mitigating the risk of animals re-ranking across different environments. Further investigations in this regard and considerations of postnatal climatic conditions were recommended by Hay and Roberts (2018). Our findings support the use of RNMs to estimate breeding values under variable EC and characterize phenotypic plasticity (Waters et al. 2024). The high proportion of robust genotypes in the investigated populations reflect opportunities to select animals with stable performances across a range of environments. To effectively capitalize on this potential, integrating EBVs from diverse climatic conditions into a composite selection index could enhance the accuracy of genetic evaluations and contribute to improved breeders’ selection decisions (Mulder 2016; Silva Neto et al. 2024). Additional selection tools, such as the use of principal component analysis or eigenfunctions from the estimated additive genetic (co)variance matrix, have been proposed to promote animal robustness without compromising productive performances (Carabaño et al. 2014; Macciotta et al. 2017). Finally, the implementation of genomic selection methods, including the identification of genetic variants related to animal robustness, offers new perspectives to upgrade breeding strategies and resilience in livestock populations affected by GxE (Braz et al. 2021; Silva Neto et al. 2024).

## Conclusion

Prenatal drought and heat stress induced genetic variations of growth performances in Namibian Simmentaler and Simbra cattle. Evidence for strong GxE impacts on maternal and direct genetic effects was found for BW and WW. Simmentaler cows exhibited stronger maternal influences against environmental stress with respective effects on BW and WW in their offspring. Conversely, Simbra cattle demonstrated lower sensitivity to environmental fluctuations. Alterations in heritability estimates and breeding values in the two breeds imply variable responses to selection and animal re-rankings depending on EC. Therefore, incorporating relevant covariates reflecting drought and heat stress into genetic evaluation models is imperative to capture GxE effects and slightly to enhance the effectiveness of breeding programs. In addition, selection for maternal robustness alongside direct productive and adaptive performances will promote herd resilience, ultimately supporting sustainable beef production in Namibian arid context.

## Supplementary Material

skag066_Supplementary_Data

## Abbreviations

BW: birth weight
CHIRPS: center infrared precipitation with station
DT: dewpoint temperature
EBV: estimated breeding values
EC: environmental conditions
GxE: genotype-environment interaction
meanTHI90: average temperature humidity index during the last 90 before calving
r_EC_: genetic correlation between the same traits along gradients of environmental conditions
r_μ_0_μ_1__: correlation between intercept and slope of additive genetic effects in the reaction norm
RH: relative humidity
RNM: reaction norm model
SB: Simbra
SM: Simmentaler
sumPrec280: cumulative precipitation during the last 280 before calving
sumPrec365: cumulative precipitation during the last 365 before calving
sumPrec90: cumulative precipitation during the last 90 before calving
T: air temperature
THI: temperature humidity index
WW: weaning weight
