# Supplementary material for: The impacts of prenatal drought and heat stress on genetic parameter estimates for birth and weaning weights in Namibian Simmentaler and Simbra cattle
Source: J Anim Sci. 2026 Feb 26;104:skag066. doi: 10.1093/jas/skag066 (PMC13023048; doi:10.1093/jas/skag066)
Supplement: skag066_Supplementary_Data [file skag066_supplementary_data.zip › Supplementary_tables.docx]

**Supplementary Table S1:** Statistics of pedigree structure and quality in Namibian Simmentaler and Simbra beef cattle

| **Statistics** | **Simmentaler** | **Simbra** |
| --- | --- | --- |
| Index of pedigree completeness (mean ± SD) | 43.4 ± 23.4 | 40.2 ± 23.6 |
| Equivalent complete generations (mean ± SD) | 2 | 1.8 |
| Equivalent complete generations (max) | 4.7 | 4.7 |
| Maximum generations traced (max) | 10 | 10 |
| Animals missing both parent information (%) | 10 | 12.8 |
| Animals missing one parent information (%) | 11.6 | 14.8 |
| Animals missing sire information (%) | 11 | 13.7 |
| Animals missing dam information (%) | 10.6 | 13.9 |

**Supplementary Table S2:** Descriptive statistics of environmental conditions in Namibian Simmentaler and Simbra beef cattle

| **Environmental**  **conditions^1^** | **min** | **max** | **Simmentaler** | |  | **Simbra** | |
| --- | --- | --- | --- | --- | --- | --- | --- |
|  |  |  | **Mean** | **SD** |  | **Mean** | **SD** |
| **sumPrec365** | 80 | 800 | 351.70 | 140.10 |  | 358.41 | 135.04 |
| **sumPrec280** | 40 | 700 | 234.50 | 137.54 |  | 252.90 | 133.16 |
| **sumPrec90** | 0 | 400 | 72.97 | 79.32 |  | 74.70 | 80.86 |
| **meanTHI90** | 53 | 73 | 65.07 | 3.72 |  | 64.87 | 3.76 |

^1^sumPrec365, sumPrec280, sumPrec90 = cumulative precipitation over 365, 280 and 90 days before birth; meanTHI90 = average temperature-humidity index over 90 days before birth

**Supplementary Table S3:** Estimates of non-genetic variance components^1^ for birth weight (BW) and weaning weight (WW) in Namibian Simmentaler and Simbra beef cattle

|  |  | Variances | | |  | Ratios (%. min – max) | | | |
| --- | --- | --- | --- | --- | --- | --- | --- | --- | --- |
|  |  | $\boldsymbol{\sigma}_{\boldsymbol{cg}}^{\boldsymbol{2}}$ | $\boldsymbol{\sigma}_{\boldsymbol{mpe}}^{\boldsymbol{2}}$ | $\boldsymbol{\sigma}_{\boldsymbol{e}}^{\boldsymbol{2}}$ |  | $\boldsymbol{\sigma}_{\boldsymbol{cg}}^{\boldsymbol{2}}$ **/** $\boldsymbol{\sigma}_{\boldsymbol{p}}^{\boldsymbol{2}}$ | $\boldsymbol{\sigma}_{\boldsymbol{mpe}}^{\boldsymbol{2}}$ **/** $\boldsymbol{\sigma}_{\boldsymbol{p}}^{\boldsymbol{2}}$ | $\boldsymbol{\sigma}_{\boldsymbol{e}}^{\boldsymbol{2}}$ **/** $\boldsymbol{\sigma}_{\boldsymbol{p}}^{\boldsymbol{2}}$ |  |
| **Simmentaler** |  |  |  |  |  |  |  |  |  |
|  | **BW** |  |  |  |  |  |  |  |  |
|  | sumPrec365 | 4.11 | 0.57 | 11.25 |  | 15.85 - 19.37 | 3.32 - 4.05 | 41.87 - 51.17 |  |
|  | sumPrec280 | 4.17 | 0.64 | 10.83 |  | 16.10 - 19.87 | 3.06 - 3.77 | 40.83 - 50.39 |  |
|  | sumPrec90 | 4.35 | 0.68 | 10.55 |  | 15.14 - 19.36 | 2.91 - 3.73 | 39.35 - 50.31 |  |
|  | meanTHI90 | 4.09 | 0.72 | 10.35 |  | 16.63 - 19.82 | 3.12 - 3.72 | 42.25 - 50.36 |  |
|  | **WW** |  |  |  |  |  |  |  |  |
|  | sumPrec365 | 769.01 | 108.46 | 440.01 |  | 38.67 - 50.52 | 5.94 - 7.77 | 22.14 - 28.93 |  |
|  | sumPrec280 | 880.22 | 103.87 | 436.88 |  | 42.87 - 50.88 | 6.26 - 7.43 | 23.65 - 28.07 |  |
|  | sumPrec90 | 757.30 | 117.39 | 418.11 |  | 42.86 - 51.77 | 6.33 - 7.65 | 23.09 - 27.89 |  |
|  | meanTHI90 | 736.46 | 113.26 | 428.14 |  | 40.83 - 52.16 | 5.90 - 7.55 | 21.46 - 27.43 |  |
| **Simbra** |  |  |  |  |  |  |  |  |  |
|  | **BW** |  |  |  |  |  |  |  |  |
|  | sumPrec365 | 1.80 | 1.00 | 11.31 |  | 7.88 - 9.25 | 2.83 - 3.32 | 40.52 - 47.57 |  |
|  | sumPrec280 | 2.09 | 0.52 | 11.07 |  | 9.67 - 10.25 | 3.14 - 3.33 | 44.85 - 47.57 |  |
|  | sumPrec90 | 2.14 | 0.82 | 11.36 |  | 7.51 - 9.37 | 2.51 - 3.14 | 37.44 - 46.74 |  |
|  | meanTHI90 | 1.77 | 0.56 | 11.01 |  | 8.58 - 10.31 | 2.71 - 3.25 | 41.31 - 49.65 |  |
|  | **WW** |  |  |  |  |  |  |  |  |
|  | sumPrec365 | 553.46 | 38.88 | 344.74 |  | 38.73 - 43.75 | 3.8 - 4.29 | 22.51 - 25.43 |  |
|  | sumPrec280 | 720.62 | 47.39 | 329.16 |  | 37.43 - 47.03 | 3.3 - 4.15 | 18.26 - 22.96 |  |
|  | sumPrec90 | 557.42 | 46.84 | 330.17 |  | 34.86 - 46.73 | 2.93 - 3.93 | 17.51 - 23.49 |  |
|  | meanTHI90 | 622.94 | 44.04 | 320.55 |  | 33.39 - 46.79 | 2.88 - 4.04 | 16.67 - 23.38 |  |

^1^$\sigma_{cg}^{2}$ = contemporary group variance, $\sigma_{mpe}^{2}$ = maternal permanent environment variance, $\sigma_{e}^{2}$ = residual variance, $\sigma_{p}^{2}$ = total phenotypic variance

**Supplementary Table S4:** Genetic correlation ($r_{\mu_{0}\mu_{1}}$) between intercept and slope for maternal additive genetic effects for birth weight (BW) and weaning weight (WW) from the reaction norm model, and slope-to-intercept variance ratio ($\boldsymbol{\sigma}_{\boldsymbol{\mu}_{\boldsymbol{1}}}^{\boldsymbol{2}}\boldsymbol{/}\boldsymbol{\sigma}_{\boldsymbol{\mu}_{\boldsymbol{0}}}^{\boldsymbol{2}}$) describing environmental sensitivity to precipitation and average temperature-humidity index conditions in Namibian Simmentaler and Simbra beef cattle

| **Environmental covariates^1^** | **Simmentaler** | | **Simbra** | |
| --- | --- | --- | --- | --- |
|  | **BW** | **WW** | **BW** | **WW** |
| $\boldsymbol{r}_{\boldsymbol{\mu}_{\boldsymbol{0}}\boldsymbol{\mu}_{\boldsymbol{1}}}$ |  |  |  |  |
| sumPrec365 | 0.15 | 0.13 | 0.35 | -0.27 |
| sumPrec280 | 0.55 | 0.51 | 0.09 | 0.14 |
| sumPrec90 | 0.47 | 0.69 | 0.52 | 0.09 |
| meanTHI90 | -0.20 | 0.26 | 0.14 | -0.40 |
| $\boldsymbol{\sigma}_{\boldsymbol{\mu}_{\boldsymbol{1}}}^{\boldsymbol{2}}\boldsymbol{/}\boldsymbol{\sigma}_{\boldsymbol{\mu}_{\boldsymbol{0}}}^{\boldsymbol{2}}$ |  |  |  |  |
| sumPrec365 | 1.91 | 1.31 | 0.57 | 0.22 |
| sumPrec280 | 1.27 | 1.03 | 0.05 | 0.43 |
| sumPrec90 | 0.69 | 0.63 | 0.41 | 0.34 |
| meanTHI90 | 1.58 | 1.59 | 0.58 | 0.29 |
