# Supplementary figures and images for: The impacts of prenatal drought and heat stress on genetic parameter estimates for birth and weaning weights in Namibian Simmentaler and Simbra cattle
Source: J Anim Sci. 2026 Feb 26;104:skag066. doi: 10.1093/jas/skag066 (PMC13023048; doi:10.1093/jas/skag066)

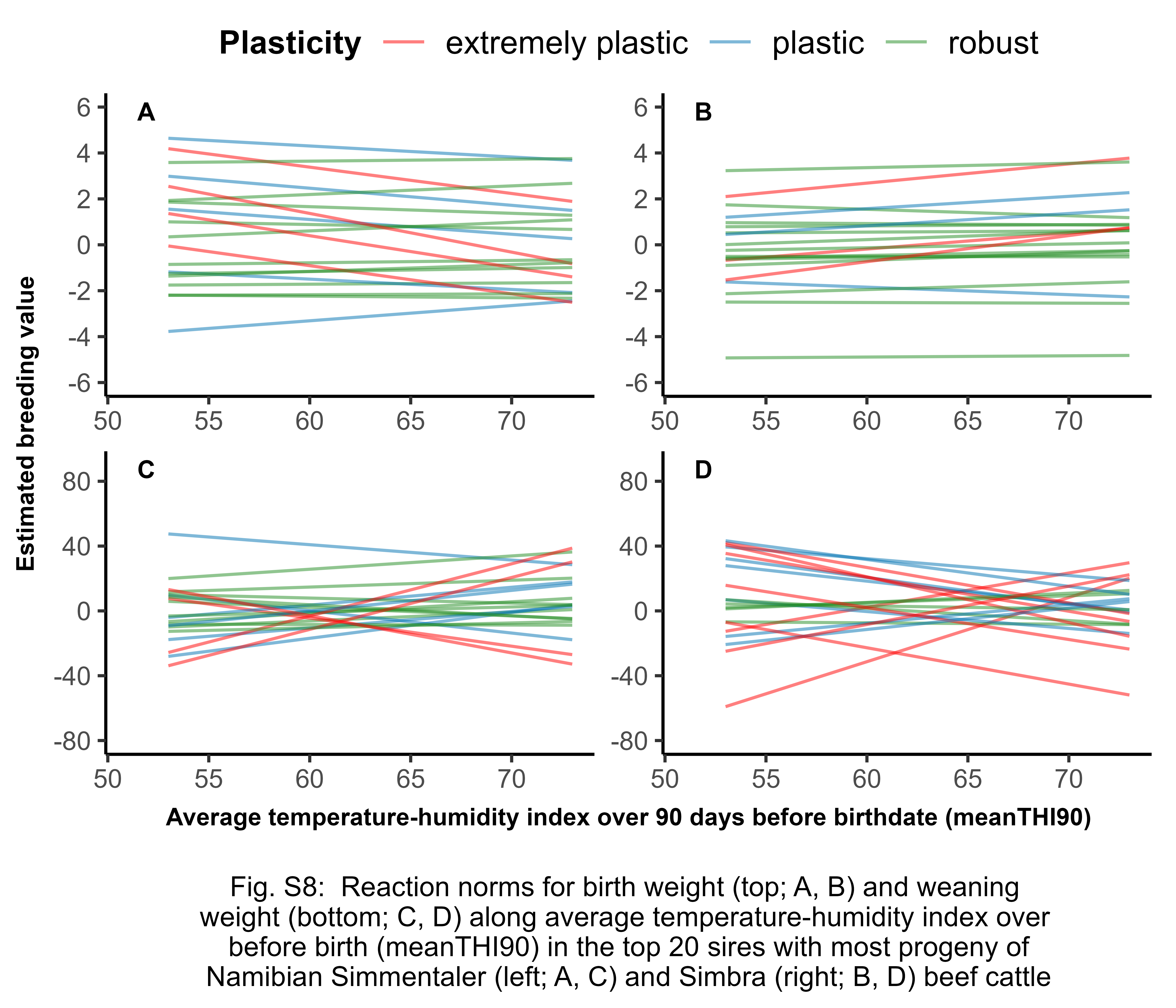

Supplement: skag066_Supplementary_Data [file skag066_supplementary_data.zip › Sup_Fig. 8.tiff]

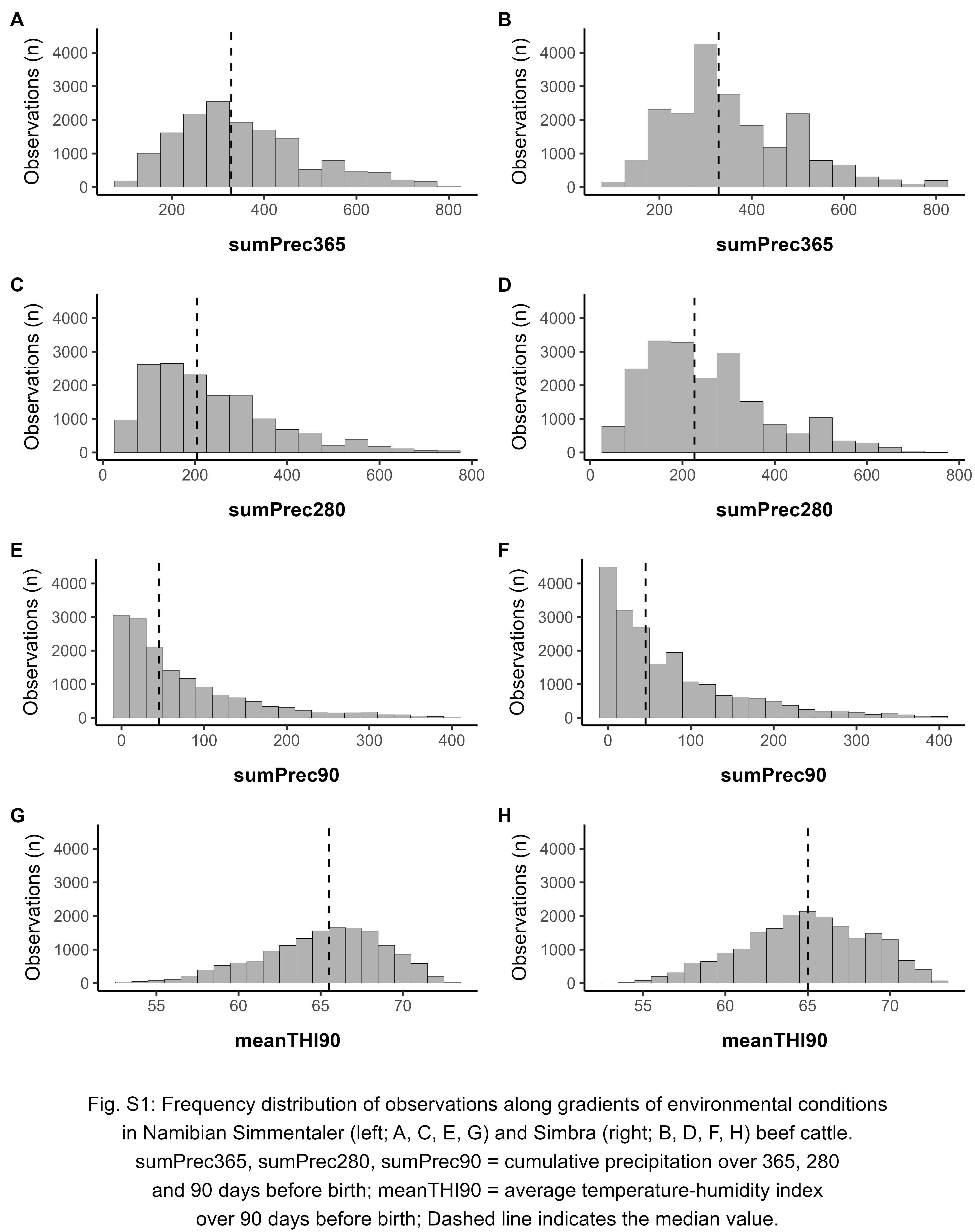

Supplement: skag066_Supplementary_Data [file skag066_supplementary_data.zip › Sup_Fig. 1.jpeg]

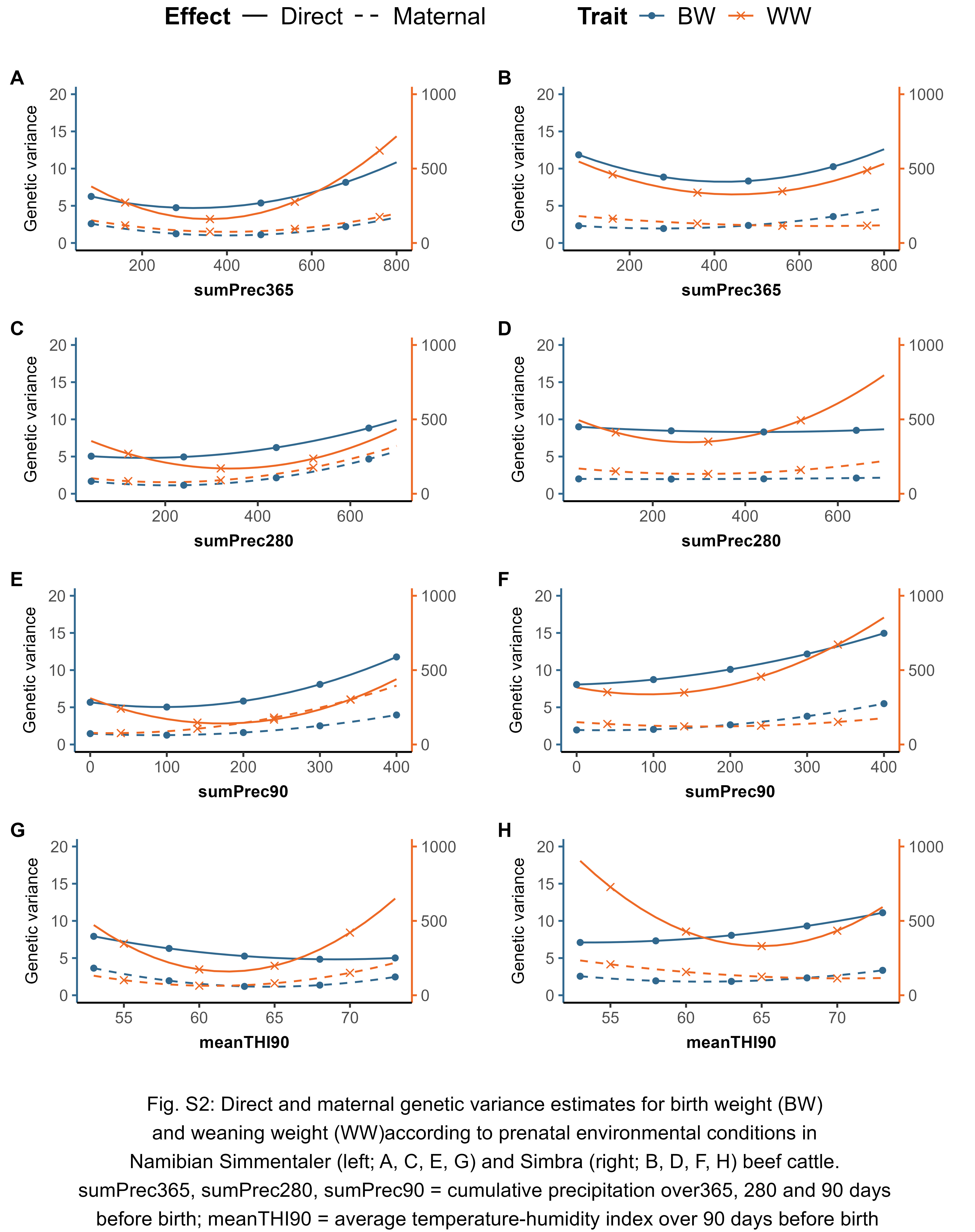

Supplement: skag066_Supplementary_Data [file skag066_supplementary_data.zip › Sup_Fig. 2.jpeg]

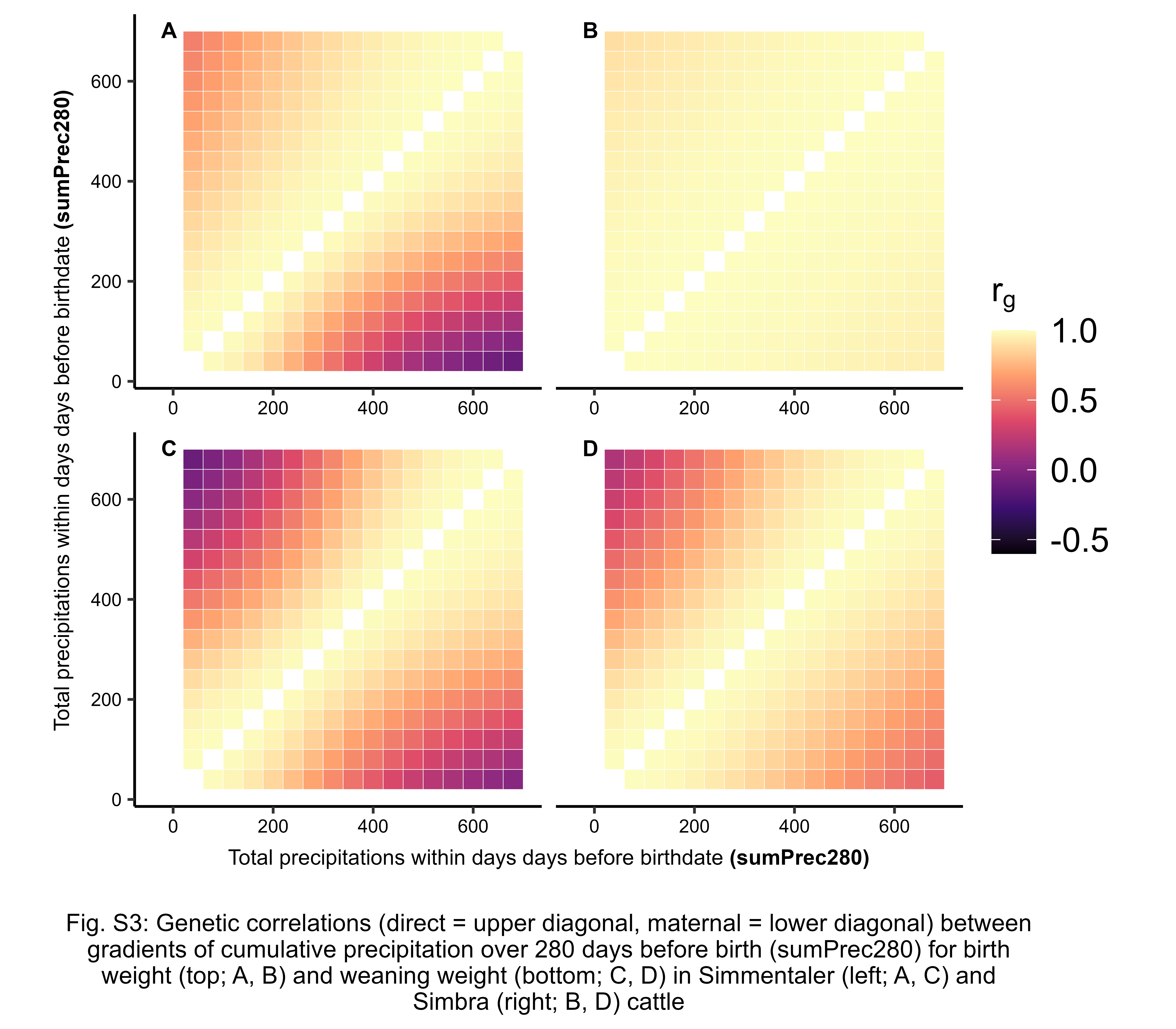

Supplement: skag066_Supplementary_Data [file skag066_supplementary_data.zip › Sup_Fig. 3.tiff]

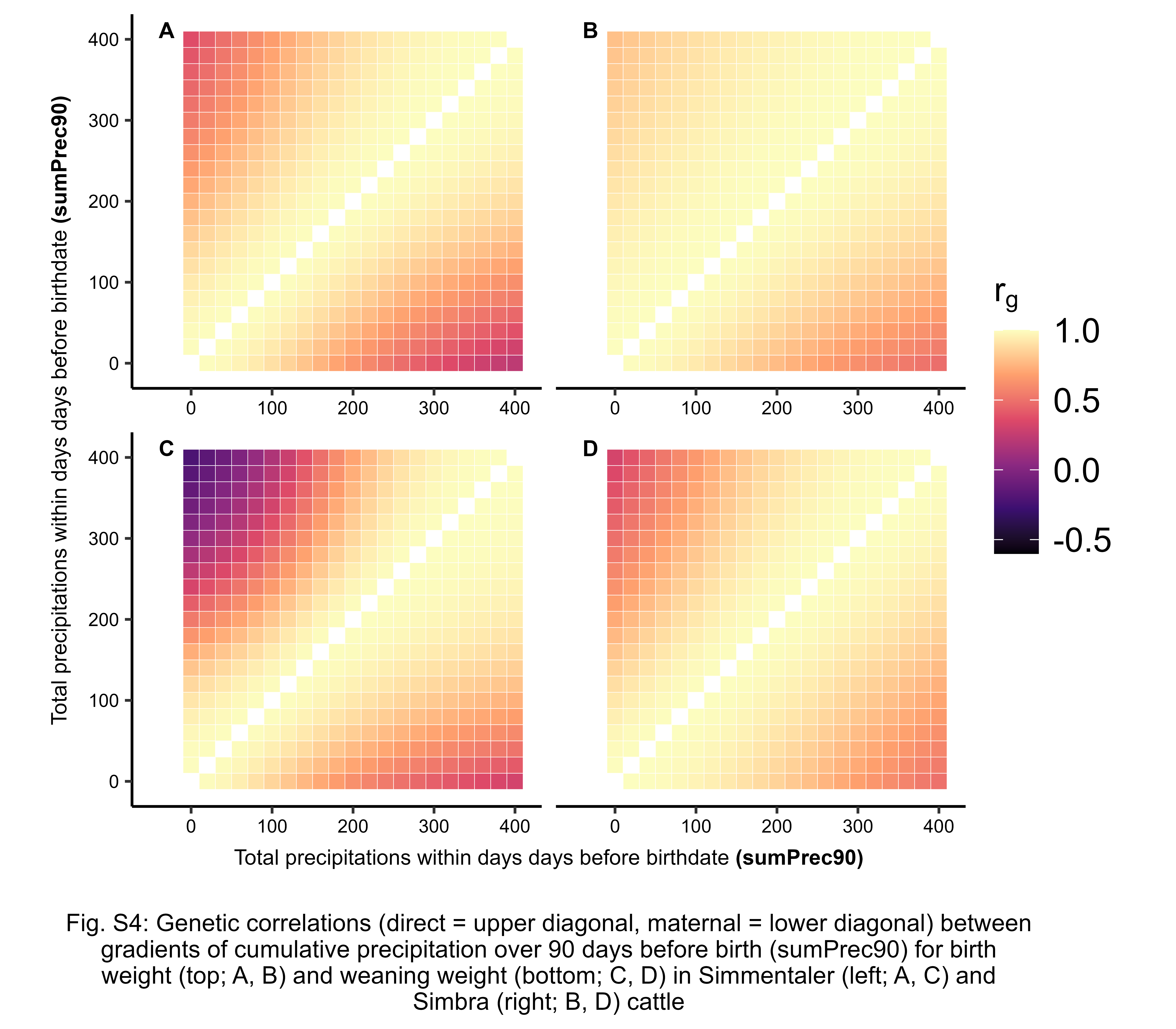

Supplement: skag066_Supplementary_Data [file skag066_supplementary_data.zip › Sup_Fig. 4.tiff]

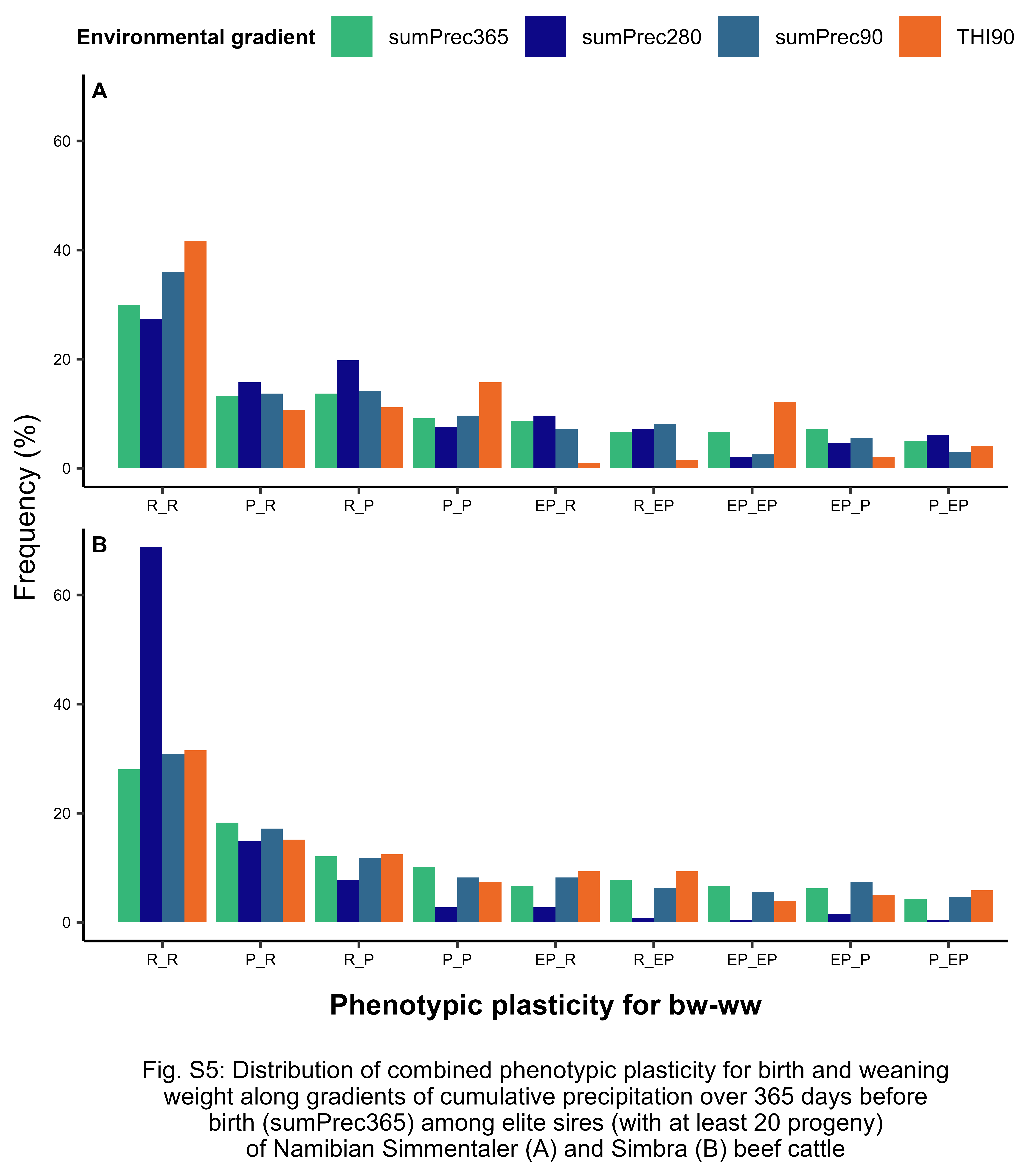

Supplement: skag066_Supplementary_Data [file skag066_supplementary_data.zip › Sup_Fig. 5.tiff]

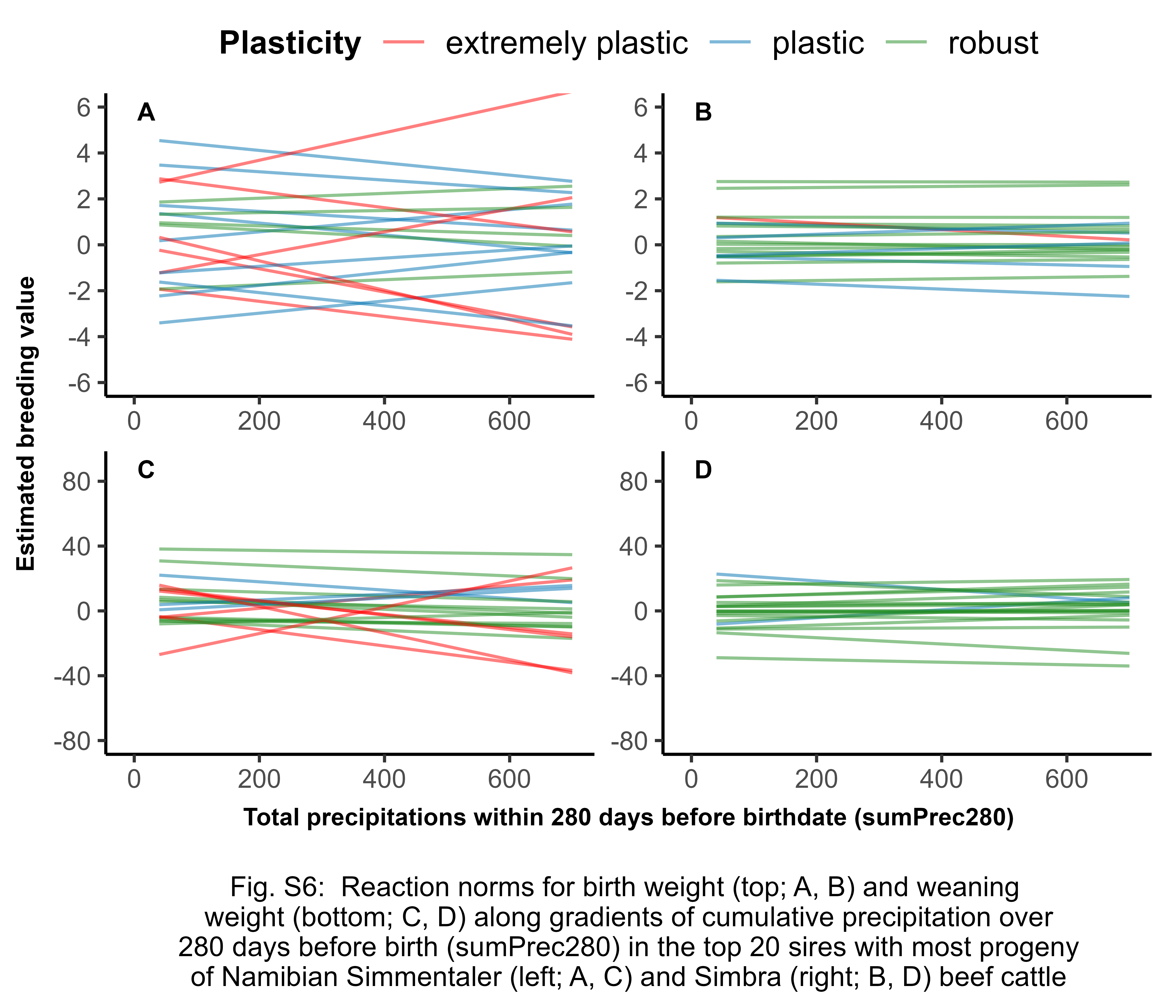

Supplement: skag066_Supplementary_Data [file skag066_supplementary_data.zip › Sup_Fig. 6.tiff]

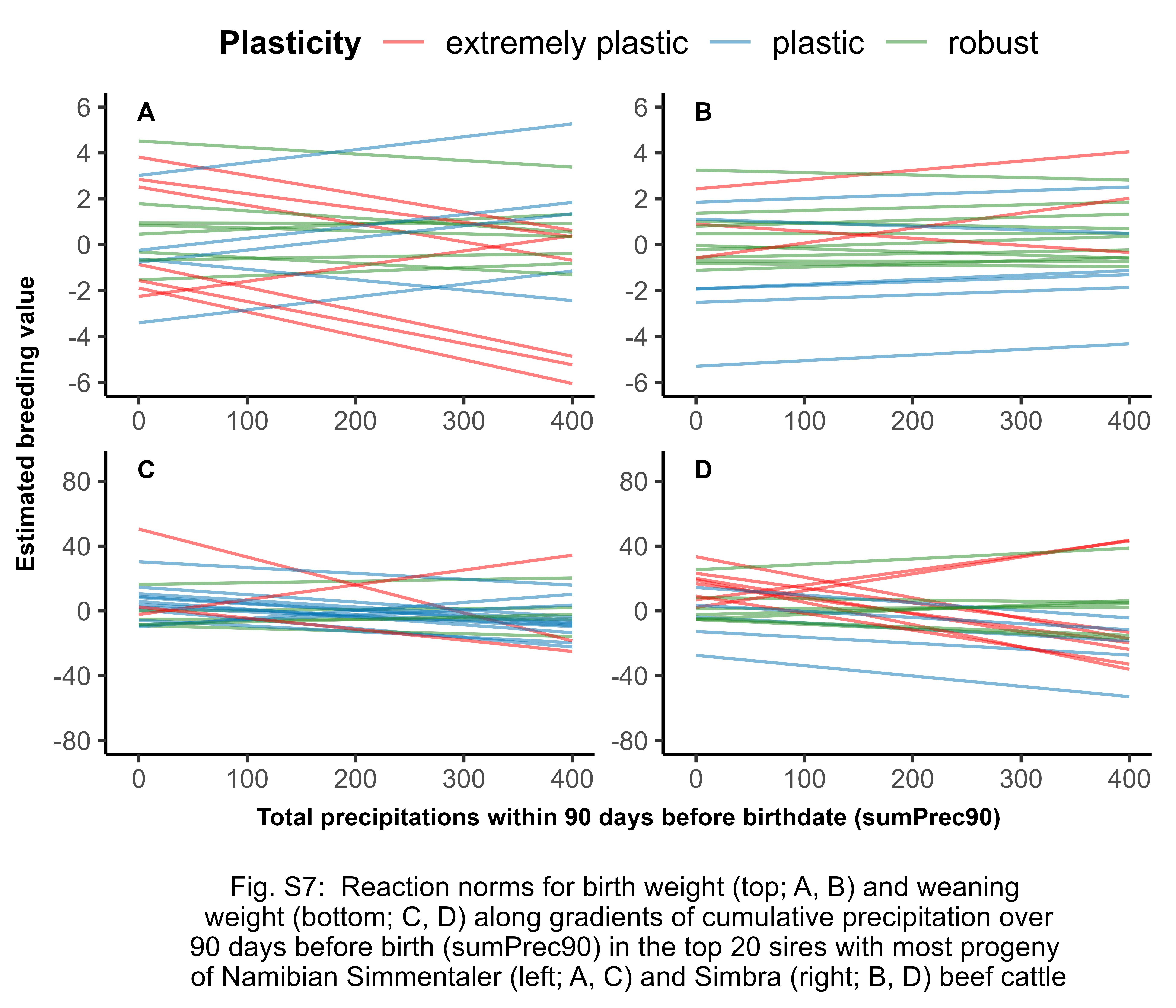

Supplement: skag066_Supplementary_Data [file skag066_supplementary_data.zip › Sup_Fig. 7.tiff]
